# Possible Association between Suicide Committed under Influence of Ethanol and a Variant in the *AUTS2* Gene

**DOI:** 10.1371/journal.pone.0057199

**Published:** 2013-02-20

**Authors:** Izabela Chojnicka, Krzysztof Gajos, Katarzyna Strawa, Grażyna Broda, Sylwia Fudalej, Marcin Fudalej, Piotr Stawiński, Aleksandra Pawlak, Paweł Krajewski, Marcin Wojnar, Rafał Płoski

**Affiliations:** 1 Department of Medical Genetics, Medical University of Warsaw, Warsaw, Poland; 2 Department of Psychiatry, Medical University of Warsaw, Warsaw, Poland; 3 Department of Forensic Medicine, Medical University of Warsaw, Warsaw, Poland; 4 Department of Cardiovascular Diseases Epidemiology, Prevention and Health Promotion, Institute of Cardiology, Warsaw, Poland; 5 Department of Psychiatry, University of Michigan, Ann Arbor, Michigan, United States of America; Oslo University Hospital, Norway

## Abstract

**Background:**

rs6943555 in *AUTS2* has been shown to modulate ethanol consumption. We hypothesized that rs6943555 might be associated with completed suicide.

**Methods:**

We genotyped rs6943555 in 625 completed suicides and 3861 controls using real-time TaqMan Allelic Discrimination Assay. All individuals were Polish Caucasians.

**Results:**

We detected an association between suicide and rs6943555 A allele (*OR* = 1.17, *P* = 0.018 for allelic comparison, *OR* = 1.24, *P* = 0.013 for dominant, and *OR* = 1.18, *P* = 0.020 for co-dominant model of inheritance). The association remained significant after adjusting for age and gender (co-dominant: *P* = 0.002 and dominant model*: P* = 0.001). After stratifying suicides according to blood ethanol concentration (BAC≤ 20 mg/dl and BAC > 20 mg/dl) the association remained significant only for cases who committed suicide under influence of alcohol (co-dominant: *OR*  =  1.37, *P* = 0.004 and dominant model: *OR* = 1.45, *P* = 0.006). To validate this finding we genotyped another cohort of 132 cases. We reproduced the association between rs6943555 A allele and suicide under influence of ethanol (allelic comparison*: OR* = 1.55, *P* = 0.023; co-dominant : *OR* = 1.54, *P* = 0.031; dominant model: *OR* = 1.84, *P* = 0.015). Analyzing pooled suicides with BAC >20 mg/dl (*N* = 300) we found the association of rs6943555 A allele not only vs. controls (allelic *OR* = 1.41, *P* = 0.00029) but also vs. cases with BAC ≤ 20 mg/dl (*N* = 449, allelic *OR* = 1.33, *P* = 0.019).

**Conclusions:**

In our study rs6943555 A allele is associated with suicide committed after drinking ethanol shortly before death. The rs6943555 A allele may be linked to adverse emotional reaction to ethanol, which could explain the association with lower consumption in general population as well as the predisposition to suicide under influence of ethanol.

## Introduction

Suicidal behavior encompasses a spectrum ranging from the suicidal thoughts to suicide attempts and completed suicide [1]. There is considerable evidence for contribution of genetic factors in suicidal behavior, both from twin [2], [3], family [4], [5] and molecular studies [6], [7]. However, the results from the genome-wide association scans suggest a role of multiple genes with small effects [8]–[11].

*AUTS2* (autism susceptibility candidate 2) is located on 7q11.22 and encodes a nuclear protein expressed mainly in developing brain [12] and amygdala (http://biogps.org/#goto=genereport&id=26053). Although the function of *AUTS2* is not known, it has been implicated in neurobehavioral disorders [13]. *AUTS2* was first associated with autism by studies of monozygotic twin pairs with an identical balanced translocation [14], [15]. Subsequently, balanced and/or unbalanced chromosomal rearrangements including *AUTS2* were found in other cases of autism [16]–[18] as well as mental retardation [19], epilepsy [20], dyslexia [13] and attention deficit hyperactivity disorder (ADHD) [21].

Recently rs6943555 in *AUTS2* has been convincingly associated (*P<<* 10^−6^) with alcohol consumption based on a genome-wide study including 12 population samples of European ancestry comprising 26 316 individuals with replication genotyping in additional 21 185 subjects [22]. The direction of the observed effect was such that the minor (ancestral) A allele was associated with 5.5% lower alcohol consumption [22]. The role of *AUTS2* in modulating alcohol consumption was further evidenced by correlations of its transcripts levels with voluntary alcohol consumption and alcohol sensitivity in mice and *Drosophila* models, respectively [22], whereas the significance of *AUTS2* variation was shown by the genotype-specific expression of this locus in human prefrontal cortex [22].

There are numerous reports linking alcohol abuse and the risk for suicide [23]–[27]. Furthermore, there is also evidence for shared predisposition for suicide and autism spectrum disorders [28], [29], including failures with social problem solving [30]–[32], lower socialization [33], deficits in emotion recognition [29], [34] and executive functioning [35]–[39].

Given these data we hypothesized that there was an association between rs6943555 and completed suicide, which could possibly be modulated by exposure to ethanol shortly before death. The aim of out study was to test this hypothesis.

## Materials and Methods

### Subjects

Suicide victims were consecutive cases from the Warsaw metropolitan area autopsied in the Department of Forensic Medicine at the Medical University of Warsaw, Poland. The information about clinical variables, such as age, gender, suicide method, blood ethanol concentration, psychiatric diagnosis and history of addiction was collected from the post-mortem medical and forensic examination protocols. Due to limited number of cases, for which definite negative history of addiction and information about psychiatric diagnosis could be obtained, we pooled those with the negative history with the cases, for which data were missing. Characteristics of suicides are shown in Table 1. We also studied a replication cohort of 134 suicides who were ascertained from after the analysis in the main cohort was completed. Characteristics of cases from the replication cohort are shown in Table 2.

**Table 1:** Characteristics of suicide subjects.

		Number of cases (%)
**Gender** * (data available for 608 cases)	Males	494 (81.3)
	Females	114 (18.7)
**Age** ** (data available for 542 cases)	mean = 43.71, SD = 16.82	542
**Blood ethanol concentration** (data available for 625 cases)	Under influence of alcohol (ethanol concentration > 20 mg/dl), mean = 185.0, SD = 110.1	235 (37.6)
	Without influence of alcohol (ethanol concentration ≤ 20 mg/dl)	390 (62.4)
**Method of committing suicide** (data available for 582 cases)	Hanging	446 (76.6)
	Jumping from a high place	68 (11.7)
	Self-harm by sharp object	17 (2.9)
	Shot with a firearm	17 (2.9)
	Jumping or lying before moving object	14 (2.4)
	Toxic effect from ingested substance	11 (1.9)
	Other	9 (1.6)
**Psychiatric disorders**	No	585 (93.6)
	Yes:	40 (6.4)
	Depression	13 (32.5)
	Schizophrenia	2 (5.0)
	Other or unknown	25 (62.5)
**History of addiction**	No	602 (96.3)
	Yes:	23 (3.7)
	Alcoholism	21 (91.3)
	Drug addiction	1 (4.4)
	Alcoholism and drug addiction	1 (4.4)

* We observed statistically significant difference regarding gender between cases and controls (47.11% males for controls, *p*<0.001 vs. suicides)

** We observed statistically significant difference regarding age between cases and controls (mean = 45.70, SD = 14.91 for controls, *p* = 0.003 vs. suicides)

**Table 2:** Characteristics of suicide subjects from the replication cohort.

		Number of cases (%)
**Gender** (data available for 131 cases)	Males	117 (89.3)
	Females	14 (10.7)
**Age** (data available for 122 cases)	mean = 42.89, SD = 16.76	122
**Blood ethanol concentration** (data available for 124 cases)	Under influence of alcohol (ethanol concentration > 20 mg/dl), mean = 157.5, SD = 86.5	65 (52.4)
	Without influence of alcohol (ethanol concentration ≤ 20 mg/dl)	59 (47.6)
**Method of committing suicide** (data available for 127 cases)	Hanging	101 (79.5)
	Jumping from a high place	14 (11.0)
	Self-harm by sharp object	3 (2.4)
	Shot with a firearm	2 (1.6)
	Jumping or lying before moving object	5 (3.9)
	Toxic effect from ingested substance	1 (0.8)
	Other	1 (0.8)
**Psychiatric disorders**	No	111 (84.1)
	Yes:	21 (15.9)
	Depression	11 (52.4)
	Schizophrenia	2 (9.5)
	Other or unknown	8 (38.1)
**History of addiction**	No	127 (96.2)
	Yes:	5 (3.8)
	Alcoholism	5 (100)
	Drug addiction	0
	Alcoholism and drug addiction	0

A special consideration was given to the use of postmortem samples from suicides from whom informed consent could not be obtained. The testing was performed using a part of blood sample routinely collected during all autopsies. All samples and phenotype data were anonimised immediately after obtaining. An approval of this protocol was obtained from the Warsaw Medical University Ethical Board (KB/185/2010).

The control group, which has been used in a previous study [40], [41] comprised subjects from the WOBASZ project - the Polish National Multicenter Health Survey – a cross-sectional study on the prevalence and control of risk factors for cardiovascular disease conducted in 2003–2005 by the Institute of Cardiology in Warsaw. The WOBASZ cohort includes a representative random sample of the Polish population aged 20–74 years (mean age = 45.72, standard deviation  =  14.91). The control group consisted of 1819 males (47.11%) and 2042 females (52.89%). All subjects filled out the questionnaire (the response rate was 74.3% and 79.3% for men and women, respectively) including among others the Beck Depression Inventory (BDI). The following classification was adopted for interpreting BDI score: BDI score >19 - moderately/severely depressed, 11< BDI score ≤ 19 - mildly depressed, BDI score ≤ 11- not depressed [42], [43].

**Figure 1:**
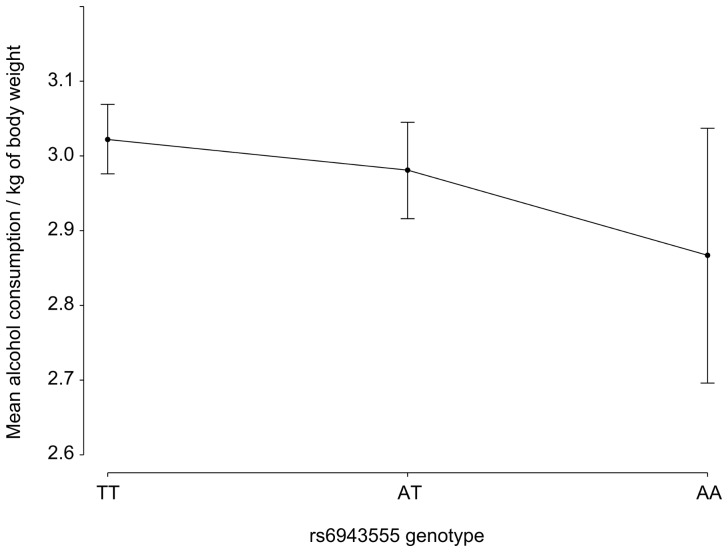
Age and sex adjusted mean quintile of alcohol consumption per day per kg of body weight vs. the rs6943555 genotype encoded as the number of the A alleles among 3830 WOBASZ subjects. Vertical bars indicate 95% confidence intervals.

The alcohol drinking habits, including the size and frequency of drinks, were obtained from a questionnaire administered by a certified interviewer as described previously [40], [41]. From these data the mean amount of ethanol consumed daily was calculated assuming that distilled spirits, wine and beer contained 316 g, 94.81g and 31.6 g of ethanol in one liter, respectively. Among studied subjects the data on alcohol consumption were available for 3830 subjects of whom 17.15% (*N* = 657) were classified as non drinkers. The median alcohol consumption per day per 1 kg of body weight among those who reported drinking alcohol was 0.027 with quartile range from 0.008 to 0.089. After adjustment for sex and age, among the WOBASZ subjects there was an inverse trend (*P* = 0.076) for correlation between the rs6943555 genotype (encoded as the number of the A alleles) and alcohol consumption expressed as quintile of amount consumed per day per kg of body weight (Fig. 1), which is consistent with previously reported results on association between *AUTS2* and alcohol consumption in a general population [22].

All cases and controls were Polish Caucasians. The study was approved by the Bioethical Committee of Medical University of Warsaw and all control subjects gave written consent for the anonymous use of their DNA for research.

### Genotyping

Genomic DNA from cases and from controls was isolated from EDTA blood samples using salting out procedure described by Miller [44]. The rs6943555 polymorphism was genotyped using Real-time TaqMan Allelic Discrimination Assay using pre-designed primers obtained from Applied Biosystems (Pre-designed TaqMan SNP Genotyping Assays, 7500 Real time PCR System, Applied Biosystems, Assay ID C____240452_20) on ABI PRISM 9700 platform (Applied Biosystems). The results were analyzed using 7500 System SDS Software (Applied Biosystems).

We successfully genotyped 625 completed suicide subjects (+ 62 undetermined samples, 91% call rate) and 3861 controls (+ 157 undetermined genotypes, 96% call rate). Our study could detect with power of 0.8 (alpha  =  0.05) an allelic association conferring OR = 1.17.

### Statistical analysis

Distribution of rs6943555 genotypes among cases and controls was compared assuming dominant, co-dominant or recessive models of effect using Web-Assotest program (http://www.ekstroem.com/ assotest/assotest.html) [45]. The most likely model of inheritance was determined by *P* value for model fit (*P_fit._*). *P_fit_* allows estimating whether given model is consistent with the distribution of genotypes among cases and controls (*P*_fit_<0.05 indicates that given model should be rejected).

Comparison of the genotype distribution after adjusting for age and gender was performed by multiple logistic regression analysis using SPSS software Release 11.5.0.

Search for genotype-phenotype correlation among suicides was performed by chi square test or Mann-Whitney U test using Statistica software version 10.0 (StatSoft, Inc., Tulsa, OK).

## Results

### Analysis of the association in the study population

The distribution of genotypes was in Hardy-Weinberg Equilibrium (HWE) among subjects and controls (Table 3). As shown in Table 3 we observed an association between suicide and rs6943555. The frequency of rs6943555 A allele among controls (21%) was consistent with frequency among populations of European ancestry (22%, http://www.ensembl.org/Homo_sapiens/Variation/Population?db=corer=7:69805523-69806523v=rs6943555vdb=variationvf=24793612) The frequency of rs6943555 A allele among cases (24%) was higher than among controls (21%, *OR* = 1.17, *P* = 0.018). The genotype distribution supported the co-dominant (OR = 1.18, *P* = 0.020, *P_fit_* = 0.381) or dominant (OR = 1.24, *P* = 0.013, *P_fit_* = 0.970), but not recessive model of inheritance (*P_fit_* = 0.018, Table 3). As the mood disorders, particularly depression, are strongly associated with suicide [46], we also compared suicides to controls without signs of depression (BDI scores **≤** 11) confirming the association (OR = 1.19, *P* = 0.017; OR = 1.19, *P* = 0.019 and OR = 1.25, *P* = 0.013 for the allelic, co-dominant and dominant model respectively, Table 3).

**Table 3:** Distribution of genotypes and analysis of the association between rs6943555 and suicide.

Cohort	rs6943555 genotypes			HWE		Model		
					Allelic comparison	Recessive	Co−dominant	Dominant
					OR (CI), *P*	OR (CI) *P, P_fit_*	OR (CI) *P, P_fit_*	OR (CI) *P, P_fit_*
	TT (%)	AT (%)	AA (%)			(AA vs. TT/AT)	(AA vs. AT vs. TT)	(AT/AA vs. TT)
Suicide victims I (*N* = 625)	359 (57.4)	232 (37.1)	34 (5.4)	0.661	1.17^1^ (1.03; 1.37), 0.018	1.16^1^ (0.79; 1.68), 0.456, 0.018	1.18^1^ (1.03; 1.36), 0.020, 0.381	1.24^1^ (1.05; 1.48), 0.013, 0.970
					1.19^2^ (1.03; 1.38), 0.017	1.17^2^ (0.80; 1.73), 0.426, 0.019	1.19^2^ (1.03; 1.38), 0.019, 0.416	1.25^2^ (1.05; 1.49), 0.013, 0.924
Suicide victims I with blood ethanol concentration > 20 mg/dl (*N* = 235)	126 (53.6)	92 (39.1)	17 (7.2)	0.971	1.38^1^ (1.11; 1.70), 0.003	1.57^1^ (0.94; 2.62), 0.105, 0.017	1.37^1^ (1.11; 1.69), 0.004, 0.770	1.45^1^ (1.12; 1.89), 0.006, 0.396
Suicide victims I with blood ethanol concentration ≤ 20 mg/dl (*N* = 390)	233 (59.7)	140 (35.9)	17 (4.36)	0.482	1.08^1^ (0.90; 1.29) 0.405	0.92^1^ (0.55; 1.52), 0.732, 0.200	1.08^1^ (0.90; 1.28), 0.411, 0.298	1.13^1^ (0.92; 1.40), 0.256, 0.493
								
Suicide victims II (*N* = 132)	73 (54.5)	51 (38.1)	8 (6.0)	0.818	1.28 (0.96–1.69) 0.089	1.30 (0.62–2.69) 0.50, 0.12	1.27 (0.96–1.68) 0.099, 0.064	1.36 (0.96–1.92) 0.089, 0.85
Suicide victims II with blood ethanol concentration > 20 mg/dl (*N* = 65)	31 (47.7)	30 (46.2)	4 (6.2)	0.352	1.55^1^ (1.06–2.27) 0.023	1.32^1^ (0.47–3.66) 0.61, 0.017	1.54^1^ (1.05–2.25) 0.031, 0.26	1.84^1^ (1.13–3.01) 0.015, 0.9
Suicide victims II with blood ethanol concentration ≤ 20 mg/dl (*N* = 59) ^3^	39 (66.1)	16 (27.1)	4 (6.8)	0.210	0.96^1^ (0.61–1.51) 0.85	1.46^1^ (0.52–4.08) 0.50, 0.42	^1^ (0.61–1.50) 0.86, 0.30	0.86^1^ (0.50–1.48) 0.60, 0.36
								
Controls (*N* = 3861)	2420 (62.7)	1258 (32.6)	183 (4.7)	0.236				
Controls BDI ≤ 11 (*N* = 2676)	1681 (62.8)	870 (32.5)	125 (4.7)	0.362				

P values <0.05 were **boldfaced** ^1^ comparison with all controls; ^2^ comparison with controls with BDI ≤ 11, HWE - Hardy-Weinberg equilibrium, ^3^for 8 subjects from suicide cohort II there were no data on blood ethanol concentration

The association between suicide and rs6943555 remained significant also after adjusting for age and gender (OR = 1.29, *P* = 0.002 and OR = 1.39, *P* = 0.001 for co-dominant and dominant model of inheritance, respectively).

### Genotype-phenotype associations

Given the previously reported relationship between rs6943555 and alcohol consumption [22], we divided suicides into two groups: cases with blood ethanol concentration over 20 mg/dl and cases with blood ethanol concentration less or equal to 20 mg/dl. Interestingly, the observed association remained significant only for cases who committed suicide under influence of alcohol (OR = 1.38, P = 0.003; OR = 1.37, *P* = 0.004 and OR = 1.45, *P* = 0.006 for the allelic, co-dominant and dominant model respectively, Table 3).

We did not find statistically significant differences in the distribution of rs6943555 genotypes among suicide after stratifying for gender (*P* = 0.293), most prevalent methods of committing suicide (*P* = 0.112 for hanging vs. other methods, *P* = 0.136 for jumping from a high place vs. other methods), age (*P* = 0.985), history of addiction (*P* = 0.253) or psychiatric diagnosis (*P* = 0.739).

### Analysis in a replication cohort

Relatively many samples from suicides (62 or 9%) had low DNA quality which precluded genotyping. This was most likely caused by low initial quality of some samples and their long storage (>5 years in some cases). Since high genotype failure rate may introduce a bias due to preferential genotyping of one allele we attempted a replication of our results using a second group of samples (*N* = 134). Since these samples were collected from recent autopsies we expected a higher rate of successful genotyping. In order to verify the DNA quality of these samples we typed them with a randomly selected real time SNP assay available in our lab (an assay for rs12936511). We obtained satisfactory results for 132 (>98.5%) samples. These 132 DNA samples were analyzed for rs6943555 and consistent with their good quality typing results were obtained in 100% of cases.

The distribution of rs6943555 genotypes in the whole second group of cases as well as after stratifying according to blood ethanol concentration is shown in Table 3 (suicide victims II). In the subset with blood ethanol concentration >20 mg/dl the frequency of the allele A was higher than among controls (*OR* = 1.55, *P* = 0.023). The genotype distribution supported the co-dominant (*OR* = 1.54, *P* = 0.031, *P_fit_* = 0.261) or dominant (*OR* = 1.84, *P* = 0.015, *P_fit_* = 0.9), but not recessive model of inheritance (*P_fit_* = 0.017, Table 3). There were no such associations among those with blood ethanol concentration ≤ 20 mg/dl (Table 3).

When we pooled all suicides with blood ethanol concentration >20 mg/dl (*N* = 300) we found that the association with the rs6943555 A allele was statistically significant when assessed vs. controls (*OR* = 1.41, *CI*: 1.17-1.70, *P* = 0.00029; *OR* = 1.53, *CI*: 1.21-1.94, *P* = 0.00044; *OR* = 1.40, *CI*: 1.17-1.69, *P* = 0.00048, for allelic comparison and comparison assuming dominant, and co-dominant model, respectively) as well as vs. cases with blood ethanol concentration ≤ 20 mg/dl. (*N* = 449, *OR* = 1.33 *CI*:. 1.04-1.69, *P* = 0.019; *OR* = 1.40 *CI*: 1.04-1.88, *P* = 0.026; *OR* = 1.34 *CI* = 1.05-1.70, *P* = 0.019; for allelic comparison and comparison assuming dominant, and co-dominant model, respectively).

## Discussion

The main novel finding from our study is the association between the A allele of rs6943555 in the *AUTS2* gene and completed suicide. We observed overrepresentation of the A alleles, AA and AT genotypes in the suicide group compared to controls. After dividing cases into those who committed suicide under influence of alcohol and those without such influence, the association was observed only among cases with blood ethanol concentration over 20 mg/dl. The association between the A allele of rs6943555 and suicide under influence of alcohol was subsequently replicated in a second cohort of cases.

The direction of the observed association of the rs6943555 A allele with suicide is opposite to the association with alcohol consumption reported previously [22]. We hypothesize that rs6943555 A allele may be linked to an adverse reaction to ethanol which would explain its association with lower alcohol consumption in general population (ref. [22] and the trend observed by us among the WOBASZ subjects) as well as the predisposition to suicide under influence of ethanol observed by us. Schumann *et al.* found increased expression of *AUTS2* in human prefrontal cortex among the A vs. T allele carriers indicating that the excessive activity of the AUTS2 protein may be responsible for these effects [22]. Furthermore, inactivation or down-regulation of the *AUTS2* homolog *tay* gene in *Drosophila melanogaster* caused reduced sensitivity to ethanol despite similar internal ethanol concentrations after ethanol exposure in mutant and wild type flies [22]. In the context of our observations this suggests a scenario where the carriers of the A allele have higher expression of *AUTS2* and are more sensitive to ethanol induced negative emotions leading to suicide. Such processes could involve impulsivity, impaired decision making and emotion dysregulation after consumption [47].

Although we show that rs6943555 is associated with suicide it is not clear whether this is a primary association or an effect of marker(s) in linkage disequilibrium. It is also possible that multiple variants in *AUTS2* have independent effects of suicide risk. Since we tested a single marker our data cannot answer this question. However, it should be emphasized that the association between rs6943555 and alcohol consumption which prompted our work emerged from an extensive genome-wide study in which ∼2,5 mln. SNPs were analyzed either directly or by imputation [22]. In particular Affymetrix 500 K which was the most frequently used chip among studies selected for meta-analysis by Schumann et al. allows to type 184 SNPs within *AUTS2* and this number is further increased by imputation procedures used [22]. Whereas the Schumann et al. do not provide a detailed list of SNPs within *AUTS2* which were analyzed Fig. S3A from the article [22] indicate that in addition to rs6943555 a substantial number of other SNPS in *AUTS2* was covered. Furthermore, in addition to mapping the primary association via known SNPs Schuman et al. also performed a screen for nonsynonymous genetic variants in the exons most proximal to rs6943555 in 200 individuals [22]. Thus, although there is clearly a need for comprehensive evaluation of all *AUTS2* variants vs. suicide risk it is possible that rs6943555 or a very closely linked SNP will remain the primary candidate.

It is increasingly recognized that pathophysiology of mental disorders is heterogenic and multifactorial [48]. Shared behavioral characteristics of many of them may be associated with shared underlying molecular mechanisms. Provided they are replicated in other populations our results should lead to a better understanding of the genetic risk factors, which play a role in the pathogenesis such psychiatric phenotypes as suicide, alcohol sensitivity and autism.
